# Superimposition of 3D maxillary digital models using open-source software

**DOI:** 10.1590/2177-6709.24.2.081-091.bbo

**Published:** 2019

**Authors:** Murilo Augusto Anacleto, Bernardo Quiroga Souki

**Affiliations:** 1 Pontifícia Universidade Católica de Minas Gerais, Programa de Pós-graduação em Odontologia (Belo Horizonte/MG, Brazil).

**Keywords:** Superposition, Digital models, Class II malocclusion, 3D imaging

## Abstract

Historically, whether for research purposes or clinical monitoring, orthodontic evaluation of dental movements has been done using plaster study models and two dimensional (2D) radiographs. However, new frontiers for the diagnosis, planning and outcome assessment of orthodontic treatments have arisen, due to the revolutionary digital tools which enable a three dimensional (3D) computerized analysis of dental movements by means of digital models. However, the software for 3D analysis are often costly, resulting in limited access to orthodontists. The present study aims to describe, through a clinical case presented to the Brazilian Board of Orthodontics and Dentofacial Orthopedics, a method for the superimposition of maxillary digital models using an open-source software to evaluate dental movements.

## Resumo

Historicamente, a avaliação ortodôntica da movimentação dentária, seja com finalidade de pesquisa, seja para monitoramento clínico, tem sido feita por meio de modelos de estudo em gesso e radiografias em duas dimensões (2D). Entretanto, com o desenvolvimento da Ortodontia digital, novas fronteiras para o diagnóstico, planejamento e avaliação dos resultados dos tratamentos ortodônticos têm surgido. Os modelos digitais possibilitam uma análise computadorizada da movimentação dentária nas três dimensões (3D); porém, os *softwares* usados para as análises 3D geralmente são onerosos e de limitado acesso aos clínicos. O presente artigo visa descrever um método de sobreposição de modelos 3D da arcada dentária superior utilizando um *software* de livre acesso para avaliar a movimentação dentária, ilustrado por um caso clínico apresentado ao Board Brasileiro de Ortodontia e Ortopedia Facial.

## INTRODUCTION

Excellence in Orthodontics depends on the careful diagnosis and evaluation of treatment results. Models and its measurements should be obtained at different time-points, and compared, in order to assess and to quantify changes associated with the orthodontic treatment. Current methods for assessing dentofacial changes along the orthodontic treatment are based on the comparison of photographs taken at different time-points, superposition of cephalograms, and also direct measurements of plaster models. Indubitably, the most commonly used method for assessing tooth movement is the superimposition of serial cephalometric radiographs. However, there are some limitations and disadvantages of two-dimensional (2D) radiographic methods, such as exposition of the patient to ionizing radiation, the overlapping of bilateral anatomical structures, the magnification of the images, impairing the identification of reference landmarks and consequently tracing errors.^1,2^

Three-dimensional (3D) assessment of the dental and occlusal changes during an orthodontic treatment has been classically performed by the analysis of plaster models. Over recent years, plaster models have been progressively replaced by digital models.^3^ Digital models have many advantages when compared to traditional ones. They do not require physical storage space, present lower cost of acquisition, and are not prone to aging degradation, breakage or loss. Intra-oral scanning (which directly generates computerized digital archives of dental arches) and the scanning of old plaster models have now gained interest by orthodontist for the model analysis and diagnosis.^4^ The accuracy of digital models has been validated, and is considered adequate.^5^

The advent of digital models opened up the possibility of evaluating dental movements by superimposing models at two or more time-points, which was not possible with traditional plaster models. Thus, 3D evaluation of teeth movements is now a reality. For such purpose, it is necessary to select intraoral reference structures for the registration (3D superimposition).^6^ ^,^ ^7^

While the use of fiduciary landmarks, lines or reference planes^8^ is well consolidated in Cephalometry, when dealing with 3D digital models, there are limitations in this aspect, due to the absence of points and validated reference regions for plaster models superposition. Several techniques have been proposed to superimpose maxillary 3D digital models.^9-14^ Some areas of the palate have been considered as stable, such as the hard palate^15^ ^,^ ^16^, palatine raphe^7^ and palatine rugaes.^12,17-19^ Maxillary digital models superposition in growing patients is critical and challenging, since growth is expected to occur in the palate and also in the region of palatine rugaes.^9,20^ However, previous studies have suggested that palatine rugae are and stable reference structures.^17,18^ Apparently, the medial two thirds of the third rugae, and the small area posterior to it presents adequate anatomical stability, being considered the gold-standard area for maxillary superposition.^9^ ^,^ ^10^ ^,^ ^12^ ^,^ ^18^ ^,^ ^21^

The superimposition of mandibular digital models is even more challenging, due to the absence of stable and reliable anatomical structures identified in dental models over the time.^22^ Some authors have suggested the use of imaging methods that combine cone beam computed tomography (CBCT) with digital models.^23,24^ However, under a biological point of view, we should not expect an absolute 3D stability of a single structure in the facial region, since, under a 3D perspective, transverse facial growth promotes lateral displacements of the entire facial skeleton during normal growth.

Commercial software for digital model analysis are available in the market and are of increasing interest by orthodontists. They have been developed with simplified layouts, and are user-friendly. However, the acquisition cost is still high, which restricts the access to them. On the other hand, there are open-source software that also allows 3D model analysis. They do not have such an easy-to-use layout, but there are free-tutorials that allow training.

Thus, the aim of this study is to describe, by means of a clinical case presented to the Brazilian Board of Orthodontics and Facial Orthopedics (BBO), a method of superimposing maxillary 3D models using an open-source software to evaluate the dental movement during the treatment of Class II malocclusion in a growing patient.

## CASE REPORT

**Figure 1:**
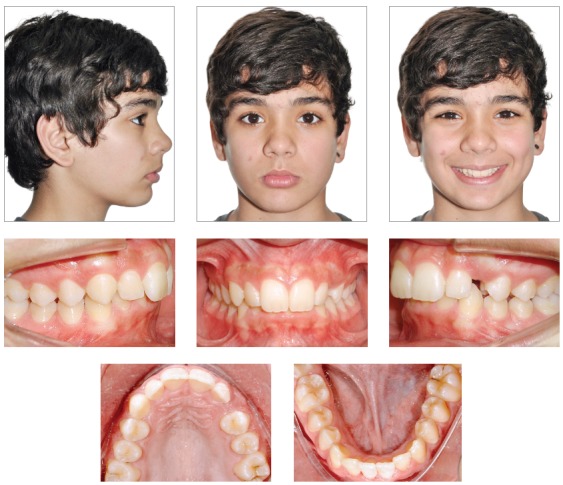
Pre-treatment facial and intraoral photographs.

A male, white-Caucasian patient, 13 years 6 months old, with good general health, sought orthodontic treatment accompanied by his mother, who reported concern about the absence of eruption of tooth #23. She pointed out the lack of room for its eruption, and that she could not see her son’s mandibular teeth. Facial clinical examination revealed a symmetric frontal view aspect, and a passive lip seal. In a sagittal view, the profile was mildly convex, the nasolabial angle was increased, with slight anteroposterior deficiency of the position of the chin (Fig 1).

**Figure 2:**
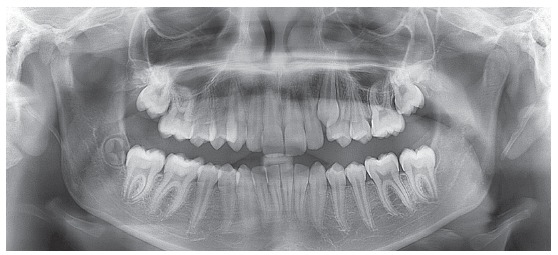
Pre-treatment panoramic radiograph.

During intraoral examination, it was observed an Angle Class II division 2 malocclusion, and a deep overbite. It was also radiographically observed an impacted tooth #23 (Fig 2). The maxillary and mandibular incisors were retroclined. Model analysis showed a negative dental discrepancy of 7 mm in the maxillary arch, and of 3 mm in the mandibular arch. Additionally, a midline deviation of 3 mm of the maxillary incisors, and a deep curve of Spee caused by the extrusion of the mandibular incisors were also diagnosed.

No mandibular deviations during mouth opening movements, no noises and symptoms of temporomandibular joint disorders were seen during functional analysis. It was verified the absence of the lateral guidance during the mandibular movements due to the impaction of the tooth #23. Analysis of the panoramic radiograph showed the obstruction for eruption of tooth #23, the presence of third molar buds, as well as normal bone trabeculae (Fig 2).

**Figure 3:**
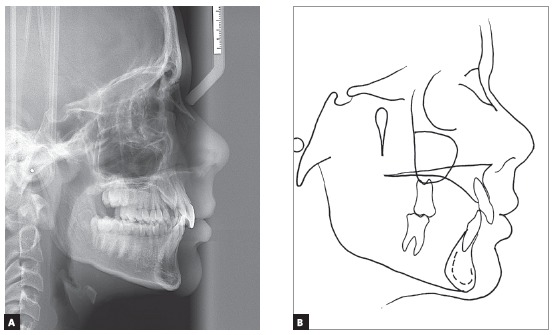
Cephalometric radiograph of profile (A) and pre-treatment cephalometric tracing (B).

Pre-treatment cephalometric analysis showed a moderate anterior-posterior discrepancy between the bone bases (ANB = 4° and Wits = 1 mm), with the maxilla slightly retruded in relation to the cranial base (ANS = 78°), the mandible moderately retruded (SNB = 74°), a high angle of facial convexity (7°) and a balanced vertical growth pattern (SN.GoGn = 31° and FMA = 21°) (Fig 3, Table 1). The Cervical Vertebrae Maturation method showed that the patient was in stage CS3, near the peak of mandibular growth.

**Table 1:** Cephalometric values. Pre-treatment (A), and (B) Post-treatment.

	Measurements		Normal	A	B	A/B diff.
Skeletal pattern	SNA	(Steiner)	82°	78°	77°	1
	SNB	(Steiner)	80°	74°	76°	2
	ANB	(Steiner)	2°	4°	1°	3
	Wits	(Jacobson)	♀ 0 ± 2 mm ♂ 1 ± 2 mm	1mm	-1mm	2
	Angle of convexity	(Downs)	0°	7°	0°	7
	Y-axis	(Downs)	59°	59°	58°	1
	Facial angle	(Downs)	87°	87°	88°	1
	SN-GoGn	(Steiner)	32°	31°	32°	1
	FMA	(Tweed)	25°	21°	20°	1
Dental pattern	IMPA	(Tweed)	90°	91°	101°	10
	1.NA (degrees)	(Steiner)	22°	17°	30°	13
	1-NA (mm)	(Steiner)	4 mm	5mm	7mm	2
	1.NB (degrees)	(Steiner)	25°	17°	28°	11
	1-NB (mm)	(Steiner)	4 mm	4mm	6mm	2
	- Interincisal angle	(Downs)	130°	143°	134°	9
	1-APo	(Ricketts)	1 mm	-1mm	2mm	3
Profile	Upper lip - S-line	(Steiner)	0 mm	1mm	0mm	1
	Lower lip - S-line	(Steiner)	0 mm	2mm	0mm	2

## TREATMENT PLANNING AND APPLIED MECHANICS 

Treatment objectives included the correction of the deep bite and the Class II relationship, the acquisition of a nice smile, with good facial balance, and occlusal functioning and stability. The proposed non-extraction treatment plan consisted of using an extraoral cervical headgear (Kloehn type), for 14 hours per day, until the correction of the Class II molar relationship. A fixed multibracket Roth prescription Edgewise appliance, slot 0.022 x 0.028-in, was used for dental leveling and alignment. Due to the increased overbite, bite ramps were made with Triad Gel^®^ light-cured resin (Dentsply GAC, York, USA) in the palatal region of the maxillary central incisors, allowing early fixed appliance installation in the mandibular arch, and the overbite correction.

The alignment and leveling were carried out in the maxillary arch with the following sequence of arches: 0.014-in NiTi; 0.018-in NiTi; 0.018-in stainless steel with omega loop tightly attached to the tubes of the first permanent molars. An open NiTi coil spring was placed between teeth #24 and #22, with elastic chain for the distalization of the premolars, thus opening space for tooth #23. After the adequate space opening, a 0.019 x 0.025-in stainless steel wire was inserted with a bypass in tooth #23, to allow for the spontaneous eruption of this tooth. Following the eruption of tooth #23 and the distalization of the posterior segments, the retraction of the maxillary incisors was carried out with a 0.019 x 0.025-in stainless steel T-loop closing loop wire.

Mandibular arch alignment and leveling were achieved with 0.014, 0.016 and 0.018-in NiTi archwires; and 0.018, 0.017 x 0.025 and 0.019 x 0.025-in stainless steel archwires. The correction of the overbite was achieved by reverting the mandibular curve of Spee, with a rectangular stainless steel archwire and resistant lingual torque in the anterior region.

Class II intermaxillary elastics were prescribed for three months. Intercuspation elastics and twist flex 0.019 x 0.025-in wire were used to finalize the treatment. Immediately after bracket debonding, a removable wraparound retainer was installed in the maxillary arch, and a 3x3 fixed retainer, manufactured with a multi-strand stainless steel 0.010 x 0.029-in archwire, was bonded to the mandibular arch.

## TREATMENT OUTCOMES

**Figure 4:**
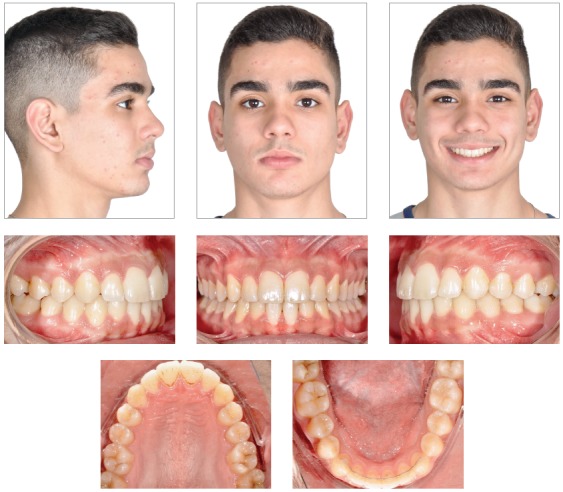
Post-treatment facial and intraoral photographs.

It was noted that the treatment goals were achieved. Improvement of the profile occurred, including facial convexity reduction, and a gain in the projection of the chin. The correction of the deep bite and the improvement of the buccal crown torque of the maxillary and mandibular incisors contributed to the harmony in the patient’s smile (Fig 4). 

**Figure 5:**
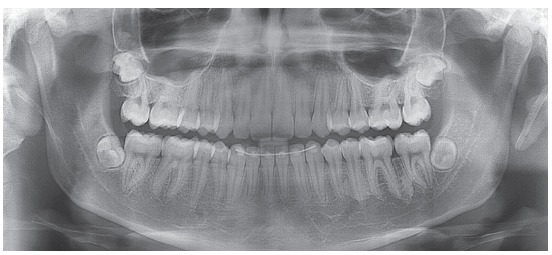
Post-treatment panoramic radiograph.

**Figure 6:**
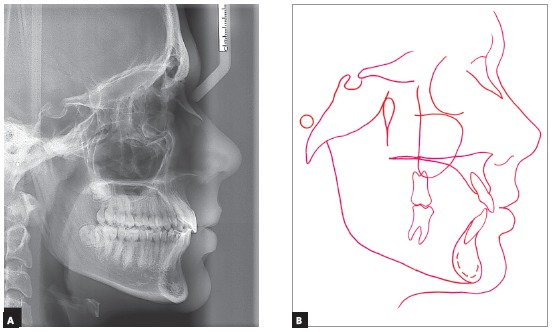
Cephalometric radiograph of profile (A) and post-treatment cephalometric tracing (B).

The deep overbite and the maxillary dental midline deviation were treated as well. Adequate molars and canines Class I relationships were obtained, with good intercuspation and appropriate arch shapes, with teeth well positioned at the bone bases, showing good root parallelism in the panoramic radiograph (Fig 5). Cephalometric evaluation showed reduction of the Facial Convexity Angle, which decreased from 7° to 0°; and also a 2-mm reduction in Wits value, from 1 mm to -1 mm. Vertical facial pattern, with a slight small backward inclination of the mandibular plane (SN.GoGn = 32°; FMA = 20°; Y axis = 58°) was maintained. In addition, the maxillary and mandibular incisors remained with adequate inclination (1.NA = 30°, 1-NA = 7 mm and 1.NB = 28°, 1-NB = 6 mm) (Fig 6, Table 1).

**Figure 7:**
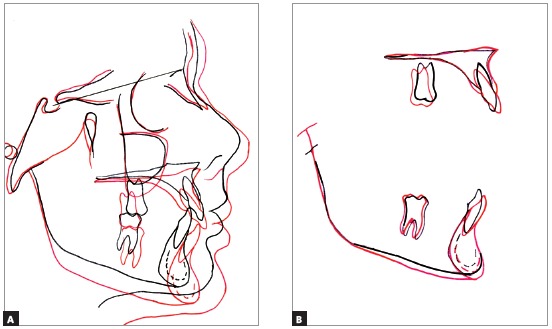
Total (A) and partial (B) superpositions of the pre-treatment (black) and post-treatment (red) cephalometric tracings.

The superposition of the cephalometric radiographs indicated considerable facial growth during the treatment period, which occurred mainly in the horizontal direction, favoring a small counterclockwise rotation of the mandible (Fig 7). Regional maxillary superposition showed that incisors were buccally flared. Molars distalization and extrusion were also identified. Regional mandible superposition revealed that molars presented slightly uprighting and extrusion; and that lower incisors crowns were also buccally tipped, but with a smaller degree than the maxillary incisors.

## 3D DIGITAL MODELS SUPERPOSITION METHOD

Several studies have performed the superposition of digital models using commercial software platforms that use simplified best-fit method.^12^ This method, available in a wide range of programs, does not consider landmarks and anatomic fiduciary regions of interest that are considered stable and valid to perform the superposition. Moreover, there is also a cost limitation in the acquisition of such programs. Therefore, this study aimed at presenting the superposition technique of maxillary digital models using an free access open-source software that allows the selection of regions of interest for its registration. 

**Figure 8:**
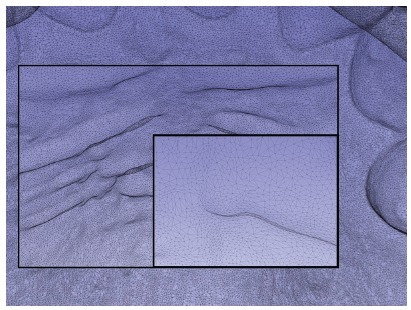
3D image of the maxillary arch showing an area of the palatine rugae where the mesh of triangles that made up the model surface can be identified.

Plaster models (pre-treatment and post-treatment) were digitized with a Smart Optical 3D Scanner^®^ (Open Technologies, Rezzato, Italy), and files were saved in stereolithography (STL) format (CMF 5.0 version, available at www.slicer.org). The superposition of the 3D models - which are made by a mesh of several triangles (Fig 8) - was performed using the 3D Slicer (CMF 5.0 version, available at www.slicer.org).

**Figure 9:**
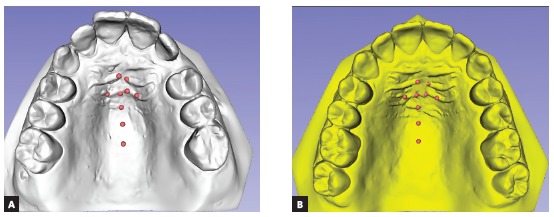
Landmarks marked in the pre-treatment (A) and posttreatment (B) models.

In order to superimpose the two maxillary digital models,^8^ nine palatal fiduciary landmarks were selected and marked by an experienced orthodontist (Figs 9A and 9B), using “Surface Registration/Add and Move Landmarks” tool, in pre-treatment and post-treatment virtual models. These points of reference were: medial points of second and third palatine rugae on both sides, midpoint between lateral and medial points of second ruga, also bilaterally; and three points on the median palatine raphe - 1) the median point of the fourth ruga; 2) 5mm posterior of the median point of the fourth ruga, 3) 10mm posterior of the median point of the fourth ruga. The selection of one landmark, actually, implies in the selection of a vertex of one of the triangles that compose the surface mesh. Since it is not possible to consistently choose the same triangle vertex in two different models, and as the triangles are not exactly of the same size, it is mandatory to improve consistency (repeatability and reproducibility) creating a working region of interest (ROI), extending the selected triangle vertex into a standardized size by the software, and thus making the two models’ ROI more symmetric.

**Figure 10:**
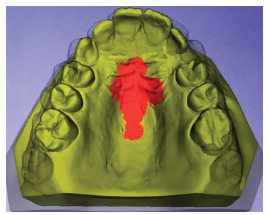
Regions of interest for the superimposition.

**Figure 11:**
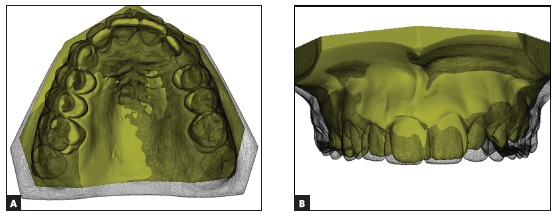
Superposition of pre-treatment (in yellow) and post-treatment (in gray) models: A) occlusal view, B) front view.

Thus, after the selection of the landmarks, a size of the ROI for each point is given to the software. A ROI of size 30 was chosen for points located on the rugae, and a ROI of size 15, for points located on the palatine raphe. A ROI of 15 means that the software will include 15 layers of triangles surrounding the triangle vertex chosen by the operator, while a ROI of 30 implies the inclusion of 30 layers around that landmark initially selected by the operator. Thus, in the present patient, an extended surface that involved the medial third of the first rugae, the 2/3 of the third rugae, and a palatine raphe area (Fig. 10) was generated. The chosen area was used as the base for the superposition of the pre-treatment and post-treatment models (Fig. 11A).

## DISCUSSION

Tools to evaluate dental movements during orthodontic treatment is of utmost importance for clinicians, including the confirmation if planned positional modifications occurred as expected, and if anchorage control was achieved. Besides that, assessing the effectiveness and efficiency of teeth movements is of interest during scientific investigations. Traditionally, this observation has been performed through the superposition of serial cephalograms, which allows the evaluation of dental movement in the sagittal and vertical perspectives. However, with the development of digital models, the superimposition of 3D models has been proposed to the evaluation of dental movements. 

In the current treatment, it was observed significant arch expansion, as well as correction of the Class II molar relationship on the right side, due to the rotation of the tooth #16. Small distalization of the tooth #26 was also noted. Moreover, digital models superposition evidenced the correction of the incisors’ cant, named as roll (rotation around the anterior-posterior axis), what can be seen on Figures 11A and 11B. Tooth rotation, maxillary expansion, and rotations can not be clearly observed in the conventional cephalometric examination.

The cephalometric regional maxillary superposition corroborates the sagittal and vertical dental changes observed in the 3D models superposition: small distalization an extrusion of the molars, buccal flaring with small extrusion of maxillary incisors. Choi et al.^25^ evaluated the validity of digital models superposition method in patients who had received treatment with rapid maxillary expansion (RME) and facemask. It was used the entire palate as the reference area for the superimposition. The measurements were compared with cephalometric findings and showed a high correlation with anteroposterior measurements, and a moderate correlation with the vertical ones. 

In the current patient’s treatment, a considerable amount of alveolar growth could be seen during the treatment period, which led to a relative deepening of the palate. The total superposition of pre-treatment and post-treatment tracings revealed a significant amount of facial growth, both sagittal and vertical. We call attention that this specific assessment can not be performed with 3D models superposition, because there is no cranial base reference for the superimposition. It is clear that 3D models superposition does not aim to eliminate the use of cephalometric tracing superposition, but rather add more information, especially the lateral tooth movement and rotations.

The proposed superposition method uses open-source software, which allows orthodontists, with no acquisition cost, to perform dental models superimposition. In addition, the majority of commercial software uses a simplified best-fit superimposition technique, in which fiduciary maxillary stable structures are not considered. One of the limitations of this free software is that it is not user-friendly, and a considerable amount of training is required for using it.

**Figure 12:**
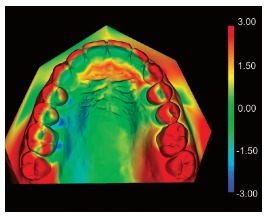
Closest-point color-map, indicating the greatest differences (displacements) between the models (in red).

Although palatal rugae are not fully stable during craniofacial growth, their instability is greater in the lateral and anterior regions.^17^ ^,^ ^18^ In the current method, the segment of the palatine rugae and the medial palatine raphe were used, considered areas with relative stability for superposition.^9^ ^,^ ^10^ ^,^ ^12^ ^,^ ^18^ ^,^ ^21^ The color-map generated demonstrated the method’s accuracy, once it was not observed any difference in superimposition areas: the medial third of the first rugae, the 2/3 of the third rugae, and the palatine raphe area (Fig 12).

Further studies on reliability and validity about this method should be performed, in order to present a standardized protocol for the digital models superposition. It is also expected that open-source software can be simplified, with a design that allow clinicians without advanced training to operate them in the daily clinical basis.

## CONCLUSION

The superimposition of 3D digital models allows interesting tooth movement analysis that can not be performed with the conventional plaster models. At the same time, the possibility of the operator selecting regions of interest in this open-source software makes more democratic the use of this new technology.
